# Distinct associations of *NEDD4L* expression with genetic abnormalities and prognosis in acute myeloid leukemia

**DOI:** 10.1186/s12935-021-02327-7

**Published:** 2021-11-22

**Authors:** Ming-qiang Chu, Liu-chao Zhang, Qian Yuan, Ting-juan Zhang, Jing-dong Zhou

**Affiliations:** 1 Department of Hematology, Affiliated People’s Hospital of Jiangsu University, 8 Dianli Rd., Zhenjiang, 212002 Jiangsu People’s Republic of China; 2 Laboratory Center, Affiliated People’s Hospital of Jiangsu University, Zhenjiang, Jiangsu People’s Republic of China; 3 Medical Laboratory, Qidong People’s Hospital, Qidong, Jiangsu People’s Republic of China; 4 Department of Oncology, Affiliated People’s Hospital of Jiangsu University, 8 Dianli Rd., Zhenjiang, 212002 Jiangsu People’s Republic of China

**Keywords:** *NEDD4L*, Expression, Prognosis, Acute myeloid leukemia

## Abstract

**Background:**

There is mounting evidence that demonstrated the association of aberrant *NEDD4L* expression with diverse human cancers. However, the expression pattern and clinical implication of *NEDD4L* in acute myeloid leukemia (AML) remains poorly defined.

**Methods:**

We systemically determined *NEDD4L* expression with its clinical significance in AML by both public data and our research cohort. Moreover, biological functions of *NEDD4L* in leukemogenesis were further tested by in vitro experiments.

**Results:**

By the public data, we identified that low *NEDD4L* expression was correlated with AML among diverse human cancers. Expression of *NEDD4L* was remarkably decreased in AML compared with controls, and was confirmed by our research cohort. Clinically, low expression of *NEDD4L* was correlated with greatly lower age, higher white blood cells, and higher bone marrow/peripheral blood blasts. Moreover, *NEDD4L* underexpression was positively correlated with normal karyotype, *FLT3* and *NPM1* mutations, but negatively associated with complex karyotype and *TP53* mutations. Importantly, the association between *NEDD4L* expression and survival was also discovered in cytogenetically normal AML patients. Finally, a number of 1024 RNAs and 91 microRNAs were identified to be linked to *NEDD4L* expression in AML. Among the negatively correlated microRNAs, *miR-10a* was also discovered as a microRNA that may directly target *NEDD4L*. Further functional studies revealed that *NEDD4L* exhibited anti-proliferative and pro-apoptotic effects in leukemic cell line K562.

**Conclusions:**

Our findings indicated that *NEDD4L* underexpression, as a frequent event in AML, was associated with genetic abnormalities and prognosis in AML. Moreover, *NEDD4L* expression may be involved in leukemogenesis with potential therapeutic target value.

**Supplementary Information:**

The online version contains supplementary material available at 10.1186/s12935-021-02327-7.

## Background

Acute myeloid leukemia (AML) is a heterogeneous clonal aggressive malignancy characterized by the uncontrolled proliferation and blocked differentiation of myeloid precursor cells [1]. Cytogenetic and genetic abnormalities in leukemic cells lead to a cascade of molecular events, which in turn cause cancer phenotype and inhibit normal hematopoiesis [2]. The genetic alterations emerging in AML has been linked to prognosis and play a crucial role in treatment strategy decision [3]. Moreover, gene expression profiling has been widely used in AML, and was also helpful in evaluating the prognostic risk and disease recurrence [4]. At the same time, accumulating studies have reported that high transcript level of *BAALC*, *MN1*, *ERG*, and *WT1* was significantly associated with poorer survival in AML [5]. Accordingly, screening and identifying additional AML-related prognostic biomarkers by high-throughput sequencing could precisely recognize higher risk AML, and finally improve the clinical outcome of AML.

The neural precursor cell expressed developmentally downregulated protein 4 (*NEDD4*) family comprises of nine members including *NEDD4*, *NEDD4-2* (*NEDD4L*), *ITCH*, *SMURF1*, *SMURF2*, *WWP1*, *WWP2*, *NEDL1*, and *NEDL2* in human, which are involved in the regulation of a variety of signaling pathways [6]. *NEDD4L* belongs to the evolutionarily conserved *NEDD4* family of ubiquitin ligases characterized by a C2 domain, 2–4 WW domains, and a C-terminal HECT-type ubiquitin ligase domain [7, 8]. *NEDD4L* is originally discovered in identifying for downregulated genes during the development of the central nervous system [7, 8]. Recently, there is mounting evidence that showed the association of *NEDD4L* expression with prognosis in diverse human cancers [9–16].

Herein, as far as we known, it was the first time to report low expression of *NEDD4L* in AML. We identified and verified that *NEDD4L* was decreased in AML, and *NEDD4L* underexpression was correlated with specific cytogenetic/genetic abnormalities of AML. Moreover, low expression of *NEDD4L* was associated with clinical outcome in cytogenetically normal AML (CN-AML). Finally, a number of 1024 mRNAs and 91 microRNAs were identified to be linked to *NEDD4L* expression in AML. Among the negatively correlated microRNAs, *miR-10a* was also discovered as a microRNA that may directly target *NEDD4L*. Further functional studies revealed that *NEDD4L* exhibited anti-proliferative and pro-apoptotic effects in leukemic cell line K562.

## Materials and methods

### CCLE

The CCLE (Cancer Cell Line Encyclopedia) database (https://www.broadinstitute.org/ccle) focuses on the gene expression, methylation, and mutation data for over 1100 types of cancer cell lines [17]. *NEDD4L* expression in cancer cell lines was firstly identified by CCLE.

### HPA

The HPA (Human Protein Atlas) database (https://www.proteinatlas.org/) focuses on proteins expression in cells, tissues, and organs [18]. *NEDD4L* expression in cancer cell lines was further identified by HPA.

### GEPIA

The GEPIA (Gene Expression Profiling Interactive Analysis) database (http://gepia.cancer-pku.cn/) focuses on analyzing the RNA sequencing expression data of 9736 tumors and 8587 normal samples from the TCGA (The Cancer Genome Atlas) and the GTEx (Genotype-Tissue Expression) projects, using a standard processing pipeline [19]. *NEDD4L* expression in 33 types of cancer patients including AML and controls was analyzed by GEPIA.

### BloodSpot

The Bloodspot (http://servers.binf.ku.dk/bloodspot/) provides a plot of gene expression in hematopoietic cells at different maturation stages based on curated microarray data [20]. *NEDD4L* expression between among AML subtypes and controls was identified by Bloodspot.

### TCGA databases

TCGA is a landmark cancer genomics program, which molecularly characterized over 20,000 primary cancers and normal samples spanning 33 cancer types. The current study included a total of 173 AML patients with RNA-sequencing data (RNA Seq V2 RSEM) from the databases of TCGA (AML NEJM 2013) downloaded by cBioportal (http://www.cbioportal.org/) [21]. Expression and mutation data of these patients were also obtained by mRNA- and DNA-sequencing. Clinical features and treatment regimens for these patients were as reported [21].

### GEO databases

Gene Expression Omnibus (GEO) is a public functional genomics data repository supporting MIAME-compliant data submissions. Three GEO datasets (GSE12417, GSE6891 and GSE10358) were used to evaluate the prognostic value of *NEDD4L* expression in AML. Firstly, the effect of *NEDD4L* expression on survival was analyzed in GSE12417 dataset which included 78 and 162 CN-AML patients through the online tool Genomicscape (http://genomicscape.com/microarray/survival.php) [22, 23]. Then, GSE6891 dataset consisted of 187 CN-AML patients as well as GSE10358 dataset comprised of 131 CN-AML patients were further used for validation.

### Patients and samples

The validation cohort of 44 AML patients at newly diagnosis time (ND-AML, used ad cases) and 47 AML patients at complete remission (CR) time (CR-AML, used as controls) was also enrolled in this study. The detailed information of 44 ND-AML patients was given in Additional file 1: Table S1. The age and sex between AML and controls presented no significant differences (*P* > 0.05). Bone marrow (BM) samples were collected from these patients. BM mononuclear cells (BMMNCs) separated from BM of these AML patients was used in this study. The current study protocol was approved by the Institutional Ethics Committee of The Affiliated People’s Hospital of Jiangsu University, and all the participants provided written informed consents.

### RNA isolation and reverse transcription

Total RNA was isolated form BMMNCs by using Trizol reagent (Invitrogen, Carlsbad, CA) as our pervious literature [24–26]. Reverse transcription was performed as reported [24–26]. The conditions performed as follows: 37 °C for 15 min, 85 °C for 5 s.

### RT-qPCR

RT-qPCR (real-time quantitative PCR) analysis was performed to detect *NEDD4L*, *CASP3* and *CASP8* mRNA using AceQ qPCR SYBR Green Master Mix (Vazyme Biotech Co., Piscataway, NJ). The primers used for *NEDD4L* expression were 5′-CCCAATAGGTTTGAAATGAA-3′ (forward) and 5′-TAGTTGTCCGTGGCAGAGTA-3′ (reverse), primers for *CASP3* expression were 5′-AATGGACCTGTTGACCT-3′ (forward) and 5′-CTGTTGCCACCTTTCG-3′ (reverse), and primers for *CASP8* expression were 5′-GAGCCAGGGTGGTTAT-3′ (forward) and 5′-CCTTTGCGGAATGTAG-3′ (reverse). Moreover, *ABL1* (housekeeping gene) expression was also detected with the primers 5′-TCCTCCAGCTGTTATCTGGAAGA-3′ (forward) and 5′-TCCAACGAGCGGCTTCAC-3′ (reverse). Relative target gene expression was calculated based on the 2^ΔCT target gene (control−sample)^ ÷ 2^ΔCT *ABL1* (control−sample)^ (2^−∆∆Ct^) formula.

### Bioinformatics analysis

Analysis of differentially expressed genes (DEGs) and microRNAs associated with *NEDD4L* in AML, and the microRNAs-mRNAs network predictions could refer to our previous study [27].

### Cell line and cell culture

Human leukemic cell lines HEL, HL60, K562, MOLM13, MV4-11, NB4, OCI, SHI-1, SKM-1, THP-1 and U937 as well as human bone marrow stromal cell line HS-5 was cultured in RPMI 1640 medium (BOSTER, Wuhan, China) containing 10% fetal calf serum (ExCell Bio, Shanghai, China) and grown at 37 °C in 5% CO_2_ humidified atmosphere.

### SiRNA transfection

Knockdown of *NEDD4L* expression used for loss-of-function experiments was done by siRNA. The si*NEDD4L* (sense strand: 5′-CCUCUGUAAUGAGGAUCAUUU-3′ and antisense strand: 5′-AAAUGAUCCUCAUUACAGAGG-3′) [28] were purchased from GenePharma (Shanghai, China). SiRNA transfection was performed using the X-tremeGENE siRNA Transfection Reagent (Roche, Basel, Switzerland) according to the manufacturer’s instructions. Transfected cells were used for experiments in 48 h after siRNA transfection.

### Cell proliferation assays

The tested cells (1 × 10^5^ cells/mL) for 2 mL per well were seeded in a 6-well plate. After culturing for 0, 1 and 2 days, cells were counted in counting board for three times, respectively.

### Cell apoptosis assays

The tested cells (2 × 10^5^ cells/ml) for 2 ml per well were seeded in a 6-well plate. After culturing for 2 days, cells were used for apoptosis assays which were performed using Annexin V PE Apop Dtec Kit (BD Pharmingen, San Diego, CA) via flow cytometry. Each experiment was repeated three times.

### Statistical analysis

Statistical analysis was accomplished by SPSS 22.0 software package. Pearson’s χ^2^/Fisher’s exact test and Mann–Whitney’s U/Kruskal–Wallis H test were used for the comparison of categorical and continuous variables, respectively. The impact of *NEDD4L* expression on leukemia-free survival (LFS)/event-free survival (EFS) and overall survival (OS) was analyzed using the Kaplan–Meier method. The receiver operating characteristic (ROC) curve and area under the ROC curve (AUC) value were applied to determined *NEDD4L* expression in distinguishing AML from controls. The statistical *P*-values were two-sided and less than 0.05 in all analyses were considered as statistically significant differences.

## Results

### Low *NEDD4L* expression associated with AML

**Fig. 1:**
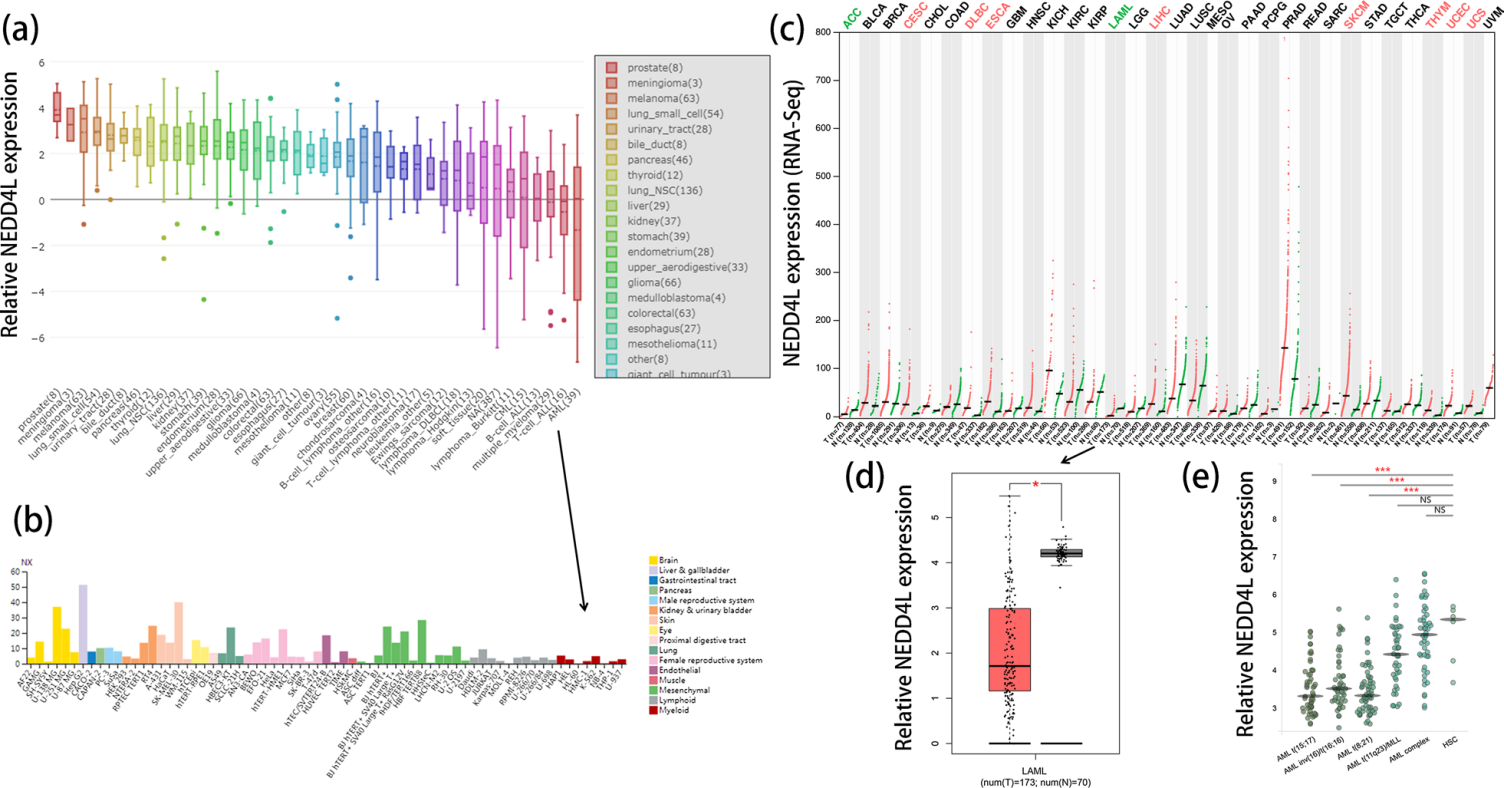
Expression of *NEDD4L* in human cancers including AML. **a** The expression of *NEDD4L* in cancer cell lines analyzed by CCLE (Cancer Cell Line Encyclopedia) dataset. **b** The expression of *NEDD4L* in cancer cell lines analyzed by the HPA (Human Protein Atlas) dataset. **c** The expression of *NEDD4L* in pan-cancer analyzed by GEPIA (Gene Expression Profiling Interactive Analysis). **d** The expression of *NEDD4L* in AML analyzed by GEPIA. *: *P* < 0.05. **e** The expression of *NEDD4L* in AML subtypes analyzed by BloodSpot

To investigate *NEDD4L* expression pattern in human cancers, we first used the CCLE databases. It was showed that *NEDD4L* was the lowest expression level in AML cell lines among 40 types of human cancer cell lines (Fig. 1a). Moreover, low *NEDD4L* expression was also closely correlated with myeloid cell lines, which was revealed by the HPA databases (Fig. 1b). Then, we further explored *NEDD4L* expression in human cancer samples and normal controls by using the GEPIA databases. Among the 33 types of human cancers, significant differences of *NEDD4L* expression between patients and controls were observed in 10 kinds of human cancers. In detail, eight of them showed increased expression, whereas two of them presented decreased expression including AML (Fig. 1c, d). Moreover, reduced expression of *NEDD4L* in AML subtypes was also showed by BloodSpot online tool (Fig. 1e). In summary, low *NEDD4L* expression was closely associated with AML among the 40 types of human cancers.

### Validation of *NEDD4L* expression in AML

**Fig. 2:**
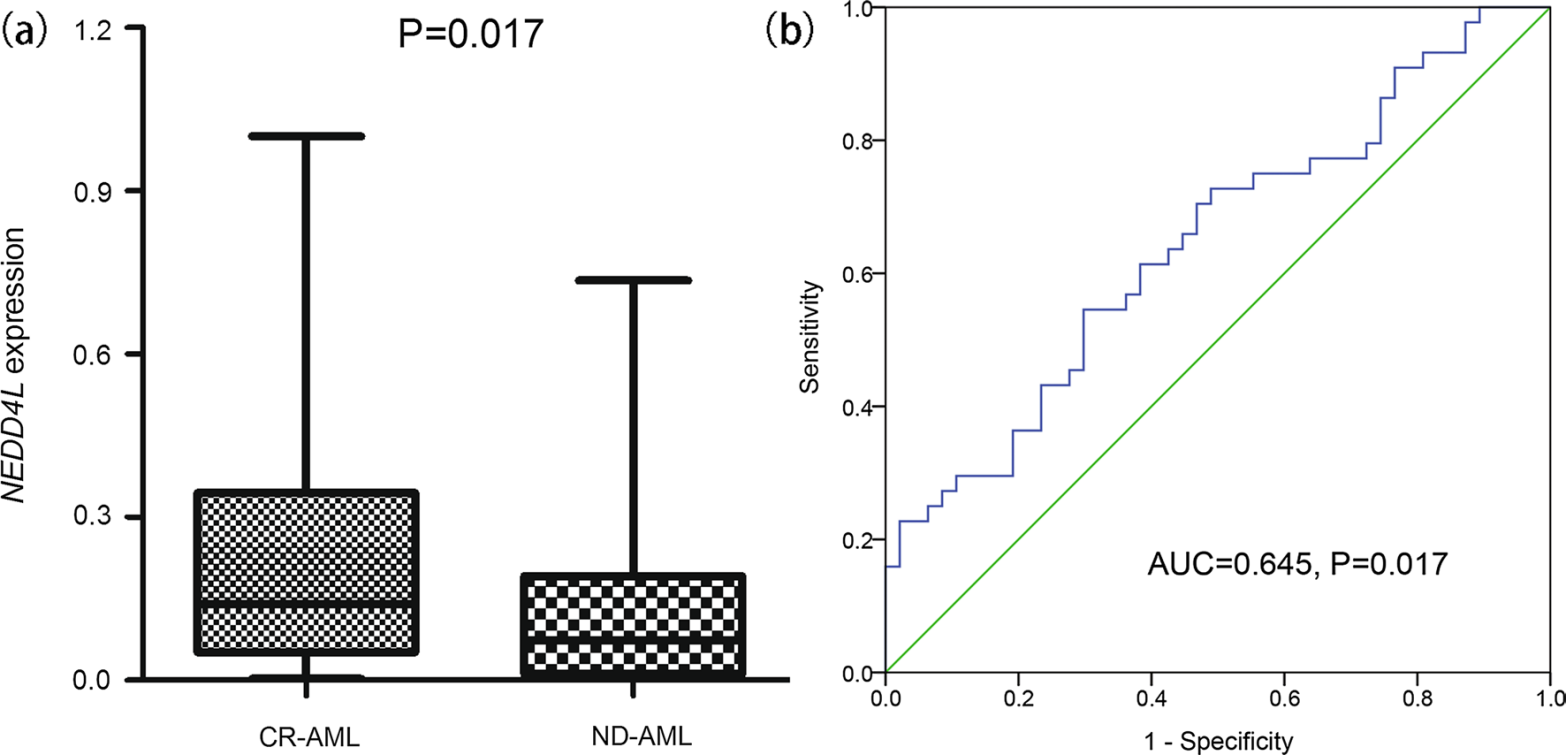
Validation of *NEDD4L* expression in AML. **a** The relative expression of *NEDD4L* in newly diagnosed AML (ND-AML) and AML achieved CR (CR-AML). Relative *NEDD4L* expression was calculated based on the 2^ΔCT *NEDD4L* (control−sample)^ ÷ 2^ΔCT *ABL1* (control−sample)^ (2^−∆∆Ct^) formula. The difference between two groups was compared by Mann–Whitney’s U test. **b** ROC curve analysis of *NEDD4L* expression in distinguishing ND-AML from CR-AML

To validate the expression pattern of *NEDD4L* expression in AML, we further detected *NEDD4L* mRNA expression in BMMNCs samples of another independent cohort of AML patients who were treated in our hospital. As expectedly, *NEDD4L* expression was significantly reduced in ND-AML (median 0.073, range 0.000–0.735) compared with CR-AML (median 0.140, range 0.003–1.000) (*P* = 0.017, Fig. 2a). Moreover, ROC analysis revealed that *NEDD4L* expression may be served as a potential biomarker for distinguishing ND-AML from CR-AML with an AUC value of 0.645 (95% confidence interval: 0.532–0.758, *P* = 0.017, Fig. 2b). These results further confirmed the low expression pattern of *NEDD4L* in AML and revealed that *NEDD4L* expression might serve as an underlying biological marker helpful for the diagnosis of AML.

### Distinct association of *NEDD4L* expression with clinical features in AML

**Fig. 3:**
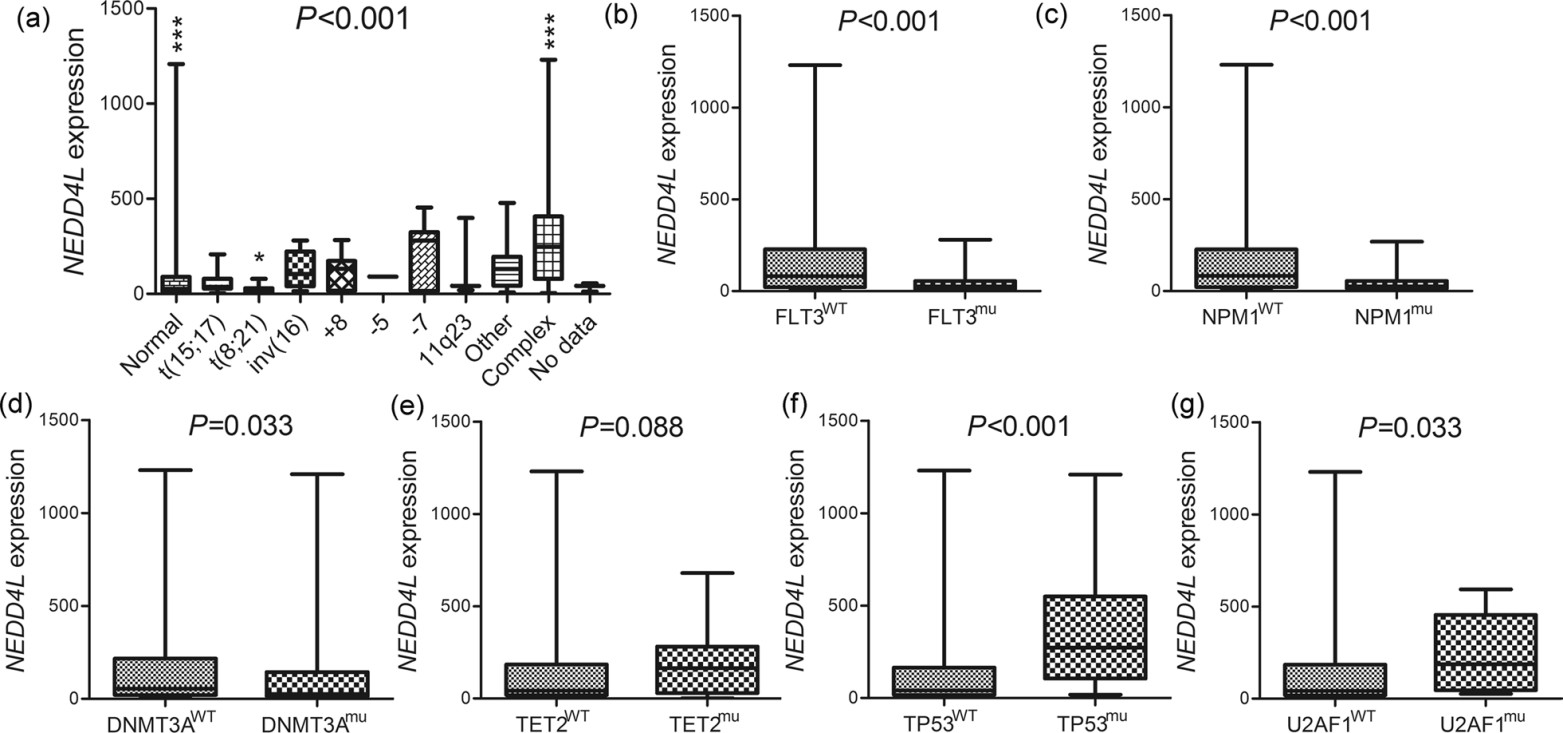
The associations of *NEDD4L* expression with cytogenetic/genetic abnormalities in AML. **a** *NEDD4L* expression among different cytogenetics of AML. *NEDD4L* expression only in normal karyotype, t(8;21), and complex karyotypes exhibited markedly difference when compared with the other karyotypes. *: *P* < 0.05; **: *P* < 0.01; ***: *P* < 0.001. **b** *NEDD4L* expression in AML patients with and without *FLT3* mutations. **c** *NEDD4L* expression in AML patients with and without *NPM1* mutations. **d** *NEDD4L* expression in AML patients with and without *DNMT3A* mutations. **e** *NEDD4L* expression in AML patients with and without *TET2* mutations. **f** *NEDD4L* expression in AML patients with and without *TP53* mutations. **g** *NEDD4L* expression in AML patients with and without *U2AF1* mutations. The difference between two groups was compared by Mann–Whitney’s U test

When analyzed the clinical implication of *NEDD4L* expression in AML, the whole-cohort cases were divided into two groups by the median level of *NEDD4L* expression. Comparison of clinic-pathologic characteristics between the two groups was presented in Table 1. AML cases with low *NEDD4L* expression exhibited markedly lower white blood cell (WBC) counts than those with high *NEDD4L* expression (*P* < 0.001). Moreover, *NEDD4L* low-expressed patients presented quite higher BM and peripheral blood (PB) blasts than *NEDD4L* high-expressed patients (*P* = 0.002 and 0.005, receptively). Moreover, significantly differences were found in the distribution of cytogenetics between low and high *NEDD4L* expressed groups (*P* < 0.001). Low *NEDD4L* expression was appreciably associated with normal karyotype (*P* = 0.001), hardly correlated with complex karyotypes (*P* = 0.001, respectively). To further exhibit the associations of *NEDD4L* expression with cytogenetic classifications, *NEDD4L* expression level among different karyotypes was further compared (*P* < 0.001, Fig. 3a). We further determined the significant associations of *NEDD4L* expression with common genetic mutations (Table 1). AML patients with low *NEDD4L* expression showed relatively higher incidence of *FLT3*, *NPM1*, and *DNMT3A* mutations (*P* = 0.007, 0.001, and 0.051 respectively) but lower frequency of *TP53*, *TET2*, and *U2AF1* mutations (*P* = 0.005, 0.063, and 0.064, respectively) than those with high *NEDD4L* expression. Moreover, the level of *NEDD4L* expression between the mutant and wild-type groups of *FLT3* (*P* < 0.001), *NPM1* (*P* < 0.001), *DNMT3A* (*P* = 0.033), *TET2* (*P* = 0.088), *TP53* (*P* < 0.001), and *U2AF1* (*P* = 0.033) genes was further exhibited (Fig. 3b–g)*.* All these results suggested that aberrant *NEDD4L* expression was correlated with diverse genetic events in AML.

**Table 1:** Correlation of *NEDD4L* expression with clinic-pathologic characteristics in AML

Patient’s parameters	*NEDD4L* expression		
	Low (n = 87)	High (n = 86)	*P* value
Sex, male/female	48/39	44/42	0.353
Median age, years (range)	55 (21–77)	61 (18–88)	0.017
Median WBC, × 10^9^/L (range)	31.5 (0.9–223.8)	8.6 (0.4–297.4)	0.000
Median PB blasts, % (range)	50 (0–97)	22 (0–98)	0.002
Median BM blasts, % (range)	76 (32–100)	62.5 (30–99)	0.005
FAB classifications			0.124
M0	7	9	0.611
M1	21	23	0.729
M2	22	16	0.359
M3	11	5	0.188
M4	13	21	0.130
M5	12	6	0.212
M6	0	2	0.246
M7	0	3	0.121
No data	1	0	1.000
Cytogenetics			0.000
Normal	51	29	0.001
t (15;17)	10	5	0.280
t (8;21)	6	1	0.117
Inv (16)	3	7	0.211
+ 8	3	5	0.496
Del (5)	0	1	1.000
−7/del (7)	2	5	0.278
11q23	2	1	1.000
Others	3	11	0.028
Complex	5	20	0.001
No data	2	1	1.000
Gene mutation			
* FLT3* (±)	33/54	16/70	0.007
* NPM1* (±)	34/53	14/72	0.001
* DNMT3A* (±)	27/60	15/71	0.051
* IDH2* (±)	9/78	8/78	1.000
* IDH1* (±)	8/79	8/78	1.000
* TET2* (±)	4/83	11/75	0.063
* RUNX1* (±)	10/77	14/72	0.388
* TP53* (±)	2/85	12/74	0.005
* NRAS* (±)	5/82	7/79	0.566
* CEBPA* (±)	7/80	6/80	1.000
* WT1* (±)	7/80	3/83	0.329
* PTPN11* (±)	4/83	4/82	1.000
* KIT* (±)	3/84	4/82	0.720
* U2AF1* (±)	1/86	6/80	0.064
* KRAS* (±)	4/83	3/83	1.000

*AML* acute myeloid leukemia, *WBC* white blood cells, *PB* peripheral blood, *BM* bone marrow, *FAB* French-American-British

### Prognostic value of *NEDD4L* expression in AML

**Fig. 4:**
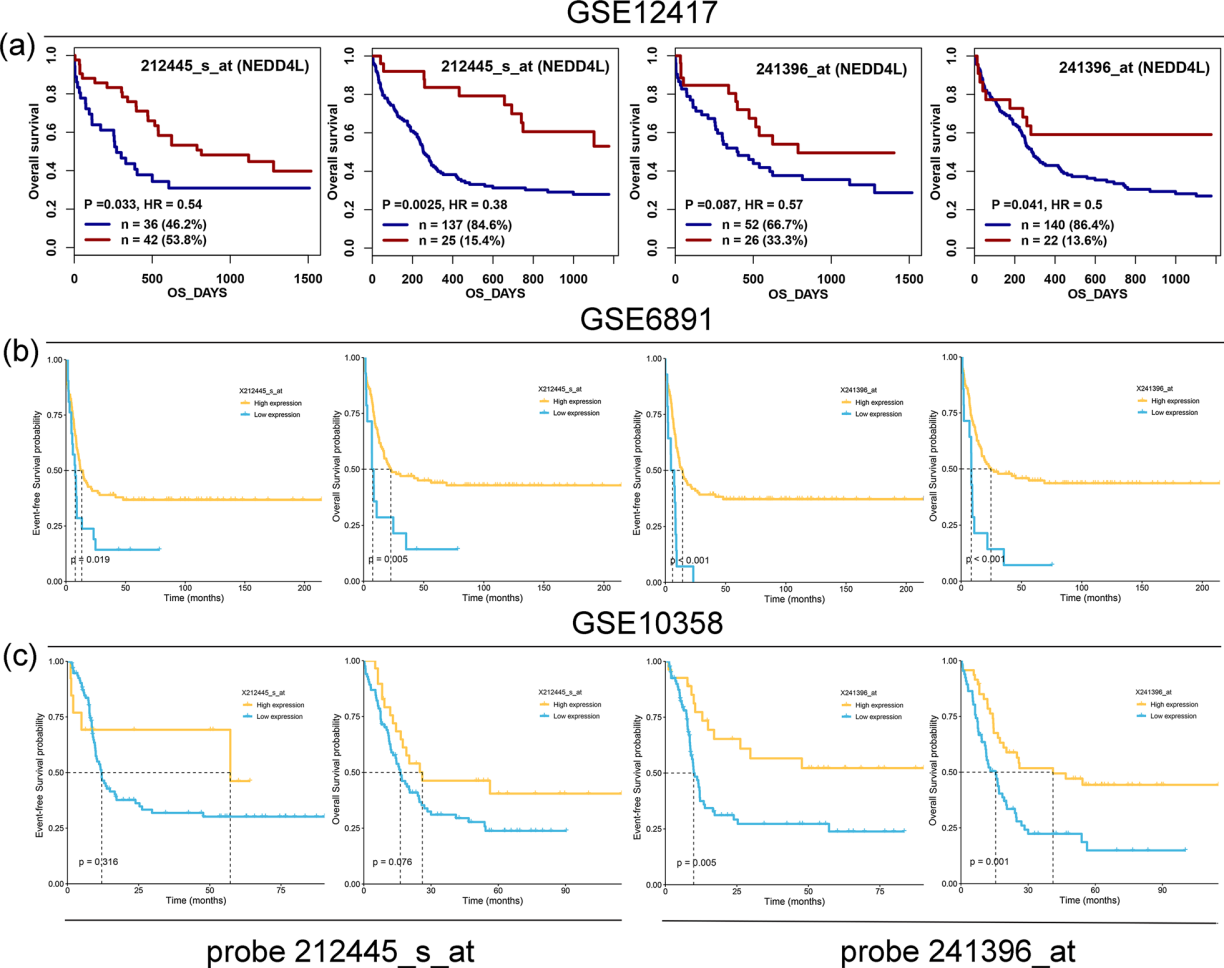
The impact of *NEDD4L* expression on survival of cytogenetically normal AML patients. **a** The effect of *NEDD4L* expression with two probes (212445_s_at and 212445_s_at) on overall survival were determined by Kaplan–Meier methods using log-rank test in two cohorts of 78 and 162 cytogenetically normal AML from the GEO dataset GSE12417. Survival analysis was performed by the online web tool Genomicscape (http://genomicscape.com/microarray/survival.php). **b** The effect of *NEDD4L* expression with two probes (212445_s_at and 212445_s_at) on event-free survival and overall survival were determined by Kaplan–Meier methods using log-rank test in 187 cytogenetically normal AML from the GEO dataset GSE6891. **c** The effect of *NEDD4L* expression with two probes (212445_s_at and 212445_s_at) on event-free survival and overall survival were determined by Kaplan–Meier methods using log-rank test in 131 cytogenetically normal AML from the GEO dataset GSE10358

We first determined the effect of *NEDD4L* expression on survival (OS and LFS) in AML from TCGA cohort. Although no remarkably differences of OS and LFS were observed between low- and high- *NEDD4L* expression groups among total AML (*P* = 0.952 and 0.972, respectively, Additional file 2: Fig. S1), patients with low *NEDD4L* expression tended to have shorter OS and LFS time than those with high *NEDD4L* expression among CN-AML (*P* = 0.161 and 0.122, respectively, Additional file 2: Fig. S1). Next, we analyzed the GEO datasets (GSE12417) including two cohorts of 78 and 162 CN-AML patients to evaluate the prognostic significance of *NEDD4L* expression in AML. The Genomicscape online tool through Kaplan–Meier analysis demonstrated that low *NEDD4L* expression was greatly correlated with shorter OS time in both 78 CN-AML (probe 212445_s_at: *P* = 0.033 and probe 241396_at: *P* = 0.087) and 162 CN-AML (probe 212445_s_at: *P* = 0.0025 and probe 241396_at: *P* = 0.041) cohorts (Fig. 4a). Moreover, the prognostic value of *NEDD4L* expression on EFS and OS was further confirmed in another two independent cohorts of CN-AML from GSE6891 (probe 212445_s_at: *P* = 0.019 and 0.005, respectively; probe 241396_at: *P* < 0.001 and 0.001, respectively) and GSE10358 (probe 212445_s_at: *P* = 0.316 and 0.076, respectively; probe 241396_at: *P* = 0.005 and 0.001, respectively) datasets (Fig. 4b, c). Taken together, low *NEDD4L* expression was correlated with unfavorable prognosis in CN-AML, and might serve as an underlying marker in AML prognosis prediction.

### Biological insights of aberrant *NEDD4L* expression in AML

**Fig. 5:**
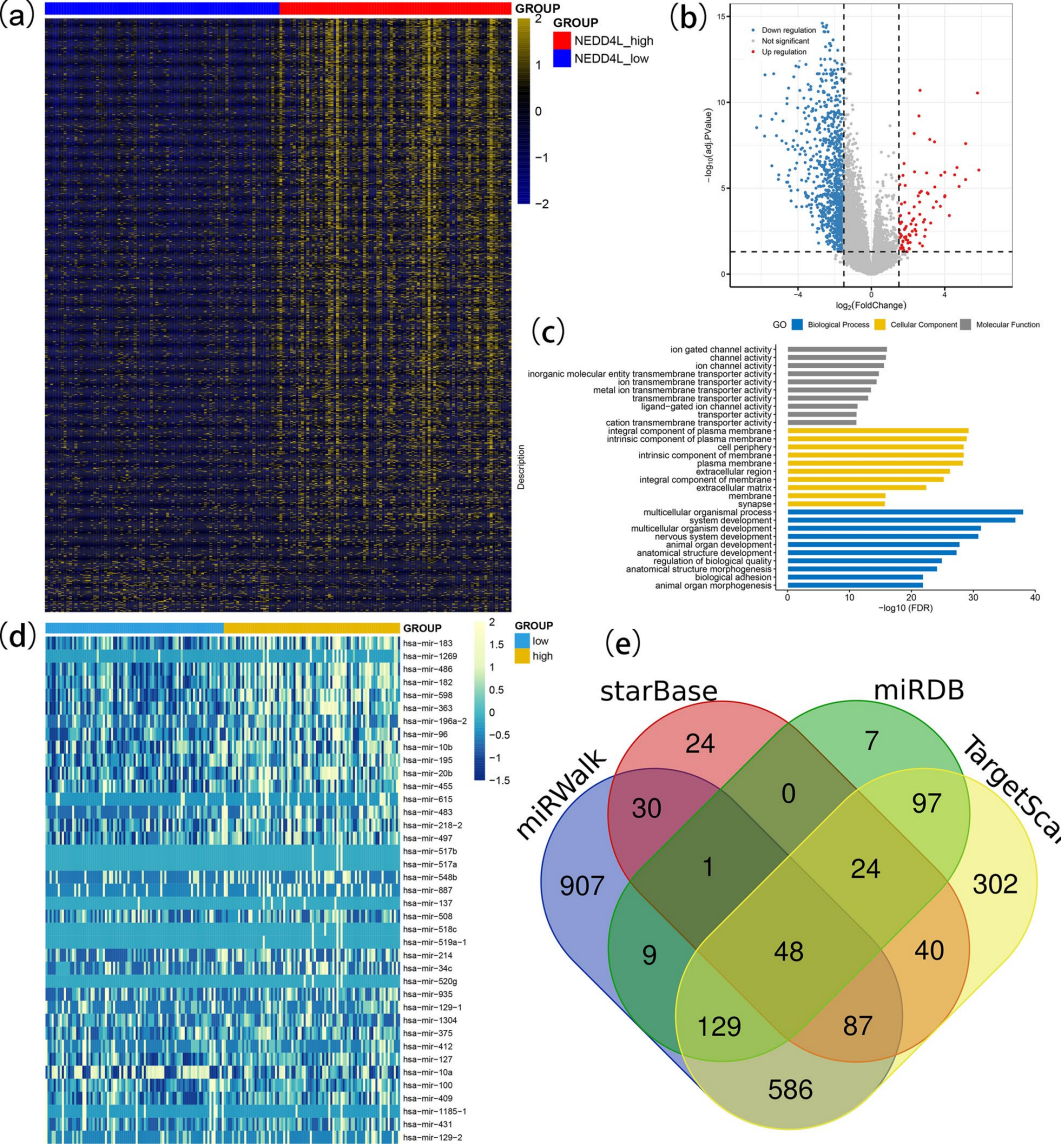
Biological insights of aberrant *NEDD4L* in AML. **a** Expression heatmap of differentially expressed genes between *NEDD4L* overexpression and underexpression groups in AML (|log2 FC|> 1.5, FDR < 0.05 and *P* < 0.05). **b** Volcano plot of differentially expressed genes between *NEDD4L* overexpression and underexpression groups in AML. **c** Gene Ontology analysis of differentially expressed genes conducted using online website of STRING (http://string-db.org). **d** Expression heatmap of differentially expressed microRNAs between *NEDD4L* overexpression and underexpression groups in AML. **e** Venn results of microRNAs which could target *NEDD4L* predicted by miRDB (http://mirdb.org/miRDB/), TargetScan (http://www.targetscan.org/vert_72/), starBase (http://starbase.sysu.edu.cn/) and miRWalk (http://mirwalk.umm.uni-heidelberg.de/)

In order to take better understanding of biological insights correlated with aberrant *NEDD4L* expression in AML among TCGA databases, we first compared the transcriptomes between high and low *NEDD4L* expression groups in AML from TCGA cohorts. A number of 1024 DEGs including 933 upregulated and 91 downregulated (high vs low) were obtained between two groups (|log2 FC|> 1.5, FDR < 0.05 and *P* < 0.05) (Fig. 5a, b and Additional file 3: Table S2). The top 50 upregulated genes including *CDH1* and the top 50 downregulated genes such as *H19* were significantly associated with prognosis in AML by our previous studies [29, 30]. In addition, the GO (Gene Ontology) analysis demonstrated that these DEGs involved in biologic processes, including multicellular organismal process, system development, multicellular organism development, and biological adhesion (Fig. 5c). Taken together, all the results supported the prognostic impact of low *NEDD4L* expression with potential role in AML.

We next determined the microRNA expression signature between low and high *NEDD4L* expression groups in AML among TCGA databases. We identified 39 differential expressed microRNAs including 27 upregulated and 12 downregulated between two groups (|log2 FC|> 1.0, FDR < 0.05 and *P* < 0.05) (Fig. 5d, Additional file 3: Table S2). Downregulated microRNAs such as *miR-375*, *miR-10a*, and *miR-100* were observed to be overexpressed in AML or have proto-leukemia effects in previous investigations [31–36]. These results together supported the anti-leukemia role and the prognostic effects of *NEDD4L* during leukemogenesis. Moreover, among these downregulated microRNAs, *miR-10a* was also discovered as a microRNA that could directly target *NEDD4L* (Fig. 5e, Additional file 4: Table S3), which indicated that *NEDD4L* may be seen as a directly target of *miR-10a* in AML*.*

### Validation of the biological role of *NEDD4L* in AML

**Fig. 6:**
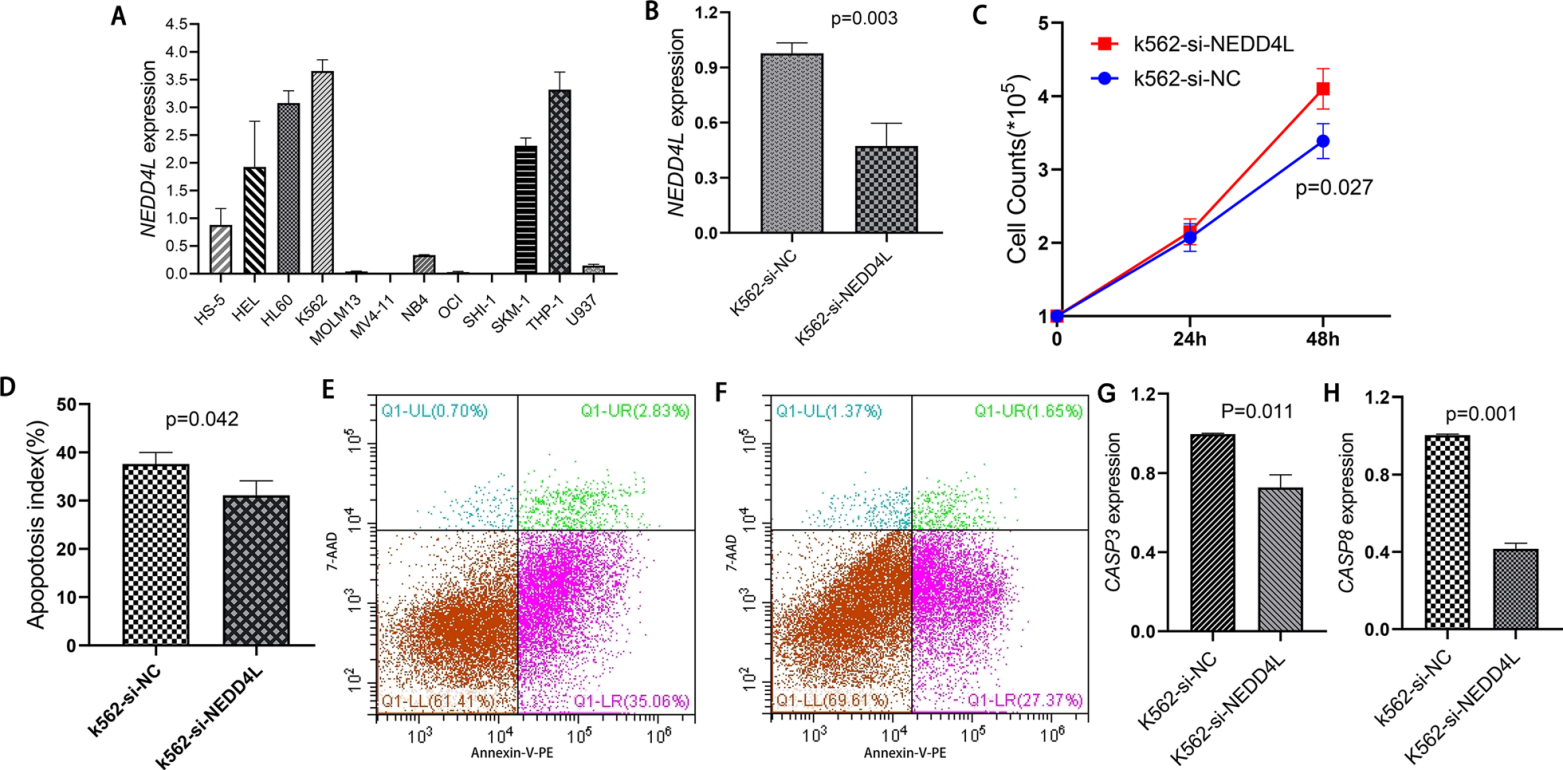
The biological role of *NEDD4L* in leukemic cell line K562. **A** *NEDD4L* expression in one human bone marrow stromal cell line and 10 common leukemic cell lines. **B** *NEDD4L* expression after siRNA-based knockdown. **C** The effect of *NEDD4L* knockdown on cell proliferation. **D** The effect of *NEDD4L* knockdown on cell apoptosis. **E**, **F** Representative flow-type apoptosis figures for K562-siNC and K562-si*NEDD4L*, respectively. **G**, **H** The effect of *NEDD4L* knockdown on the apoptosis-related gene *CASP3* and *CASP8* expression

To validate the potential role of *NEDD4L* in AML development, we next performed in vitro experiments in leukemic cells. Since it is difficult to successfully transfect *NEDD4L* that has too long coding sequence (CDS > 2000 bp) into suspension cells, we conducted loss-of-function assays in the highest *NEDD4L*-expressed cells K562 (Fig. 6A). The successfully knockdown of *NEDD4L* expression in K562 cells by siRNAs was confirmed through RQ-PCR (Fig. 6B). Expectedly, K562-si*NEDD4L* cells presented markedly increased proliferation rate (Fig. 6C) and decreased apoptosis rate as compared with K562-siNC cells (Fig. 6D–F). Moreover, apoptosis-related markers *CASP3* and *CASP8* were remarkably reduced after *NEDD4L* knockdown in K562 cells (Fig. 6G and H). All these results together suggested that *NEDD4L* may play a tumor suppressive role in AML biology.

## Discussion

In the current investigation, we for the first time explored *NEDD4L* expression in AML, and demonstrated that low *NEDD4L* expression was a frequent event in AML. Moreover, *NEDD4L* expression was appreciably link to the clinical outcome of CN-AML. Although it is the first report regarding the prognostic significance of *NEDD4L* expression in AML, several studies have shown the great correlations of *NEDD4L* expression with clinical outcome in solid tumors [9–16]. Reduced expression of *NEDD4L* correlated with adverse prognosis in non-small cell lung cancer, gastric cancer, hepatocellular carcinoma, ovarian cancer, and malignant glioma [9–16]. In addition, we also determined the potential role of *NEDD4L* in AML by further functional study validation, and showed the anti-proliferative and pro-apoptotic effects of *NEDD4L* in leukemic cell line K562, which suggested that *NEDD4L* may play a tumor suppressive role in AML biology. However, only a few studies determined the direct role of *NEDD4L* in tumorigenesis [10]. Accordingly, further clinical and functional studies are required to explore the potential role of *NEDD4L* in AML occurrence and development.

Additionally, we also observed a markedly correlation of *NEDD4L* expression with cytogenetic/genetic classifications in AML by our studies. Underexpression of *NEDD4L* was observed to be correlated with normal karyotype, *FLT3* and *NPM1* mutations, but negatively associated with complex karyotype and *TP53* mutations. Notably, a recent study also showed that abnormal *NEDD9* expression, a member of *NEDD* family, was highly correlated with specific French-American-British (FAB) subtypes and karyotypes as well as genetic mutations, which was similar to our results [37]. These results together disclosed that *NEDD4L* underexpression play a key role in CN-AML biology caused by genetic mutations. Future studies are needed to determine the potential associations of aberrant *NEDD4L* expression with genetic abnormalities in CN-AML.

Accumulating studies have reported the expression of *NEDD4L* was regulated by microRNAs during biological process including cancer development. For instance, *miR-98* by directly targeting *NEDD4L* played a key role in alleviating renal fibrosis in diabetic nephropathy [38]. *MiR-494* inhibited the TGF-beta1/Smads signaling pathway and prevented the development of hypospadias through targeting *NEDD4L* [39]. Chen et al. demonstrated that IGF-1-enhanced *miR-513a-5p* signaling desensitized glioma cells to temozolomide through targeting the *NEDD4L*-inhibited Wnt/beta-catenin pathway [40]. The *miR-106b-25* cluster through the direct repression of *NEDD4L* mediated breast tumor initiation by the activation of NOTCH1 signaling [41]. Moreover, Zhu et al. reported that the E3 ubiquitin ligase *NEDD4*/*NEDD4L* was directly regulated by *miR-1* [42]. In this study, as far as we know, it is the first time to report the negative correlation of *NEDD4L* expression with *miR-10a* in AML. Although luciferase assays were not conducted to verify the direct link between *miR-10a* and *NEDD4L*, an increasingly number of studies revealed the oncogenic role of *miR-10a* with prognostic value in AML [32–34]. All the literatures in turn supported the association of *NEDD4L* with *miR-10a* together with prognostic value in AML.

## Conclusions

In summary, our findings demonstrated that *NEDD4L* underexpression, as a frequent event in AML, was associated with genetic abnormalities and prognosis in AML. Moreover, *NEDD4L* expression may be involved in leukemogenesis with potential therapeutic target value.

## Supplementary Information

**Additional file 1: Table S1.** Clinic-pathologic characteristics of AML in our research cohort.

**Additional file 2: Figure S1.** The impact of *NEDD4L* expression on survival of AML patients from TCGA cohort. The effects of *NEDD4L* expression on leukemia-free survival and overall survival were determined by Kaplan–Meier methods using log-rank test in both total AML and CN-AML patients.

**Additional file 3: Table S2.** Differentially expressed RNAs and microRNAs between low and high *NEDD4L* expression groups.

**Additional file 4: Table S3.** Venn results of microRNAs targeting *NEDD4L*.

## Data Availability

All the data involved in this study had been included in the manuscript. The public data and the several datasets used and/or analyzed during the current study are available from the corresponding author on reasonable request.

## Abbreviations

AML: Acute myeloid leukemia
NEDD4: Neural precursor cell expressed developmentally downregulated protein 4
FAB: French-American-British
CCLE: Cancer Cell Line Encyclopedia
HPA: Human Protein Atlas
GEPIA: Gene Expression Profiling Interactive Analysis
TCGA: The Cancer Genome Atlas
GTEx: Genotype-tissue expression
CN-AML: Cytogenetically normal AML
GEO: Gene Expression Omnibus
ND-AML: AML at newly diagnosis time
CR: Complete remission
CR-AML: AML at complete remission time
BM: Bone marrow
BMMNCs: BM mononuclear cells
RT-qPCR: Real-time quantitative PCR
DEGs: Differential expression genes
LFS: Leukemia-free survival
EFS: Event-free survival
OS: Overall survival
ROC: Receiver operating characteristic
AUC: Area under the ROC curve
WBC: White blood cell
GO: Gene ontology
CDS: Coding sequence
