# Supplementary material for: Distinct associations of NEDD4L expression with genetic abnormalities and prognosis in acute myeloid leukemia
Source: Cancer Cell Int. 2021 Nov 22;21:615. doi: 10.1186/s12935-021-02327-7 (PMC8607698; doi:10.1186/s12935-021-02327-7)
Supplement: Supplementary file 1 — Additional file 1: Table S1. Clinic-pathologic characteristics of AML in our research cohort. [file 12935_2021_2327_MOESM1_ESM.docx]

**Supplementary Table S1. Clinic-pathologic characteristics of AML in our research cohort**

| Patient's parameters | Clinic-pathologic characteristics |
| --- | --- |
| Sex, male/female | 27/17 |
| Median age, years (range) | 57.5 (18-93) |
| Median WBC, ×10^9^/L (range) | 16.6 (0.7-528.0) |
| Median hemoglobin, g/L (range) | 74 (33-133) |
| Median platelets, ×10^9^/L (range) | 38 (4-447) |
| BM blasts, % (range) | 56.5 (6.0*-97.5) |
| FAB subtypes |  |
| M1 | 3 |
| M2 | 19 |
| M3 | 5 |
| M4 | 12 |
| M5 | 4 |
| M6 | 1 |
| Karyotypes |  |
| normal | 20 |
| t(8;21) | 5 |
| t(15;17) | 5 |
| 11/q23 | 1 |
| +8 | 2 |
| -5/5q- | 1 |
| complex aberrant | 4 |
| others | 3 |
| No data | 3 |
| CR (-/+) | 23/21 |

AML: acute myeloid leukemia; WBC: white blood cells; BM: bone marrow; FAB: French-American-British classification; CR: complete remission. *: AML patients less than 20% BM blasts often with typical cytogenetics such as t(15;17).
