# Supplementary figures and images for: Distinct associations of NEDD4L expression with genetic abnormalities and prognosis in acute myeloid leukemia
Source: Cancer Cell Int. 2021 Nov 22;21:615. doi: 10.1186/s12935-021-02327-7 (PMC8607698; doi:10.1186/s12935-021-02327-7)

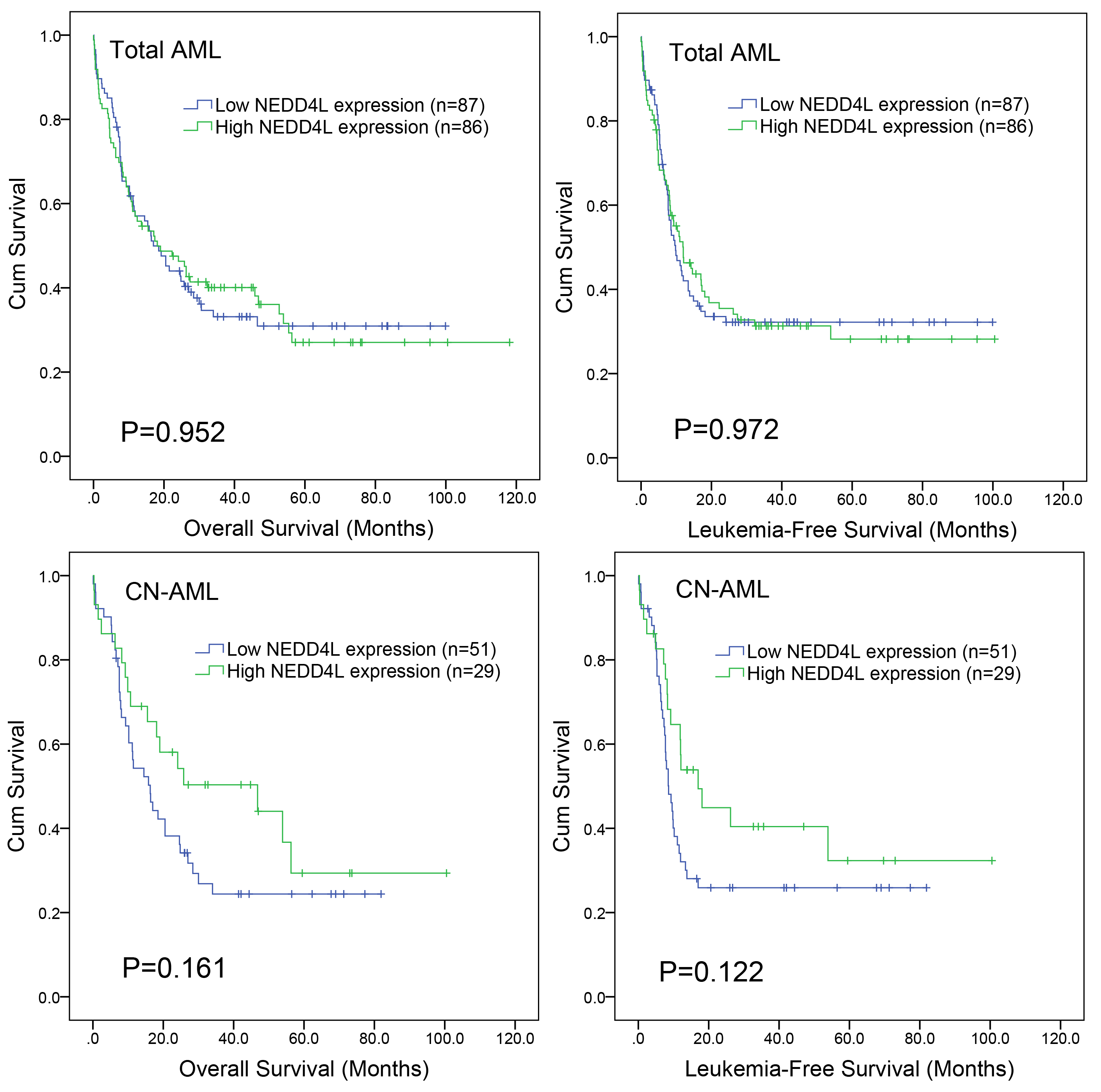

Supplement: Supplementary file 2 — Additional file 2: Figure S1. The impact of NEDD4L expression on survival of AML patients from TCGA cohort. The effects of NEDD4L expression on leukemia-free survival and overall survival were determined by Kaplan–Meier methods using log-rank test in both total AML and CN-AML patients. [file 12935_2021_2327_MOESM2_ESM.tif]
